# Anti-Inflammatory, Antioxidant, Antibiotic, and Cytotoxic Activities of *Tanacetum vulgare* L. Essential Oil and Its Constituents

**DOI:** 10.3390/medicines4020034

**Published:** 2017-05-25

**Authors:** Héloïse Coté, Marie-Anne Boucher, André Pichette, Jean Legault

**Affiliations:** 1 Laboratoire d’Analyse et de Séparation des Essences Végétales, Université du Québec à Chicoutimi, 555 boulevard de l’Université, Chicoutimi, QC G7H 2B1, Canada; Heloise.cote@uqac.ca (H.C.); Marieanne.boucher@uqac.ca (M.-A.B.); Andre.Pichette@uqac.ca (A.P.); 2 Chaire de recherche sur les agents anticancéreux d’origine naturelle, Université du Québec à Chicoutimi, 555 boulevard de l’Université, Chicoutimi, QC G7H 2B1, Canada

**Keywords:** *Tanacetum vulgare*, essential oil, GC, anti-inflammation, antioxidant, antibacterial, cytotoxicity

## Abstract

**Background:** *Tanacetum vulgare *L. (Asteraceae) is a perennial herb that has been used to treat multiple ailments. Regional variability of the chemical composition of *T. vulgare* essential oils is well-known. Despite these regional chemotypes, most relevant studies did not analyze the complete chemical composition of the *T. vulgare *essential oil and its constituents in relation to their biological activities. Here, we assess the anti-inflammatory, antioxidant, antibacterial, and cytotoxic activities of *T. vulgare *collected from northern Quebec (Saguenay-Lac-St-Jean), Canada. **Methods: **Essential oil was extracted from plants by steam distillation and analyzed using GC-FID. Biological activities of essential oil and its main constituents were evaluated in vitro. **Results: **We identified the major compounds as camphor, borneol, and 1,8-cineole. The oil possesses anti-inflammatory activity inhibiting NO production. It also inhibits intracellular DCFH oxidation induced by tert-butylhydroperoxide. Anti-inflammatory activity of essential oil appears driven mainly by α-humulene while antioxidant activity is provided by α-pinene and caryophyllene oxide. Essential oil from *T vulgare *was active against both *Escherichia coli* and *Staphylococcus aureus* with camphor and caryophyllene oxide responsible for antibacterial activity. Finally, *T. vulgare* essential oil was slightly cytotoxic against the human healthy cell line WS1 while α-humulene and caryophyllene oxide were moderately cytotoxic against A-549, DLD-1, and WS1. **Conclusion: **We report, for the first time, links between the specific compounds found in *T. vulgare* essential oil and anti-inflammatory, antioxidant, antibacterial, and cytotoxic activities. *T. vulgare* essential oil possesses interesting biological properties.

## 1. Introduction

*Tanacetum vulgare* L. is a perennial herb of the Asteraceae family. This plant grows widely in Europe and Asia. Introduced into North America in the 18th century, it is well adapted to northern climates [1]. Traditionally, *T. vulgare* has been used as an anthelmintic, tonic, emmenagogue, antihypertensive, carminative, antispasmodic, antidiabetic, diuretic, and an anti-inflammatory compound [2,3]. Most alcohol extracts of *T. vulgare* are reported to have biological effects including anti-inflammatory, antioxidant, and antibacterial properties. Indeed, studies on rats and mice [4,5] have found *T. vulgare* extracts to have anti-inflammatory properties. Flavonoids present in the plant could be, in part, responsible for these biological effects [5,6]. Shinella et al. demonstrated that parthenolide was the main in vivo anti-inflammatory substance in *T. vulgare* [5]. Methanol extracts from *T. vulgare* showed antioxidant activity, probably due to the presence of phenolic compounds [7]. The antibacterial properties of ethanolic extracts have also been reported in studies using *Escherichia coli* and *Staphylococcus aureus* [8,9]. 

Regional variability of the chemical composition of *T. vulgare* essential oils is well-known and many essential oil chemotypes have been reported in the literature [10,11,12,13,14,15,16,17,18,19,20,21]. For example, *T. vulgare* essential oil chemotypes from Hungary and Argentina are mainly composed of β-thujone (72.4% and 91.65%, respectively) [15,20]. However, *T. vulgare* essential oil chemotypes from Finland and Lithuania [10,12] had, respectively, artemisia ketone (55.08% to 81.36%) and 1,8-cineole (3.6% to 39.7%) as the main constituents. Kumar et al. [22] have thoroughly reviewed this chemotype variability of *Tanacetum*. Despite the many regional chemotypes, many studies of the biological effects of *T. vulgare* essential oils did not analyze the complete chemical composition of the oils nor its constituents in relation to their biological properties. 

In our study, the essential oil of *T. vulgare*, collected from an area in northern Quebec (Saguenay-Lac-St-Jean, Chicoutimi, QC, Canada), was extracted by steam distillation and analyzed using GC-FID (Agilent Technologies, Santa Clara, CA, USA). We assessed the essential oils and its constituents for anti-inflammatory, antioxidant, antibacterial, and cytotoxic activities.

## 2. Experimental Section

### 2.1. Chemicals

We used standard compounds for GC analyses and biological testing from Sigma-Aldrich (St. Louis, MO, USA) (1,8-cineole, bornyl acetate, α-terpinene, β-pinene, α-pinene, γ-terpinene, ρ-cymene, camphene, camphor, caryophyllene oxide, limonene, terpinene-4-ol, terpinolene), Fluka (Fluka Chemie GmbH, Buchs, Switzerland) (α-humulene), and Takasago (Takasago International Corporation, Tokyo, Japan) (β-caryophyllene).

### 2.2. Plant Material and Extraction of Essential Oil

The aerial portions of *T. vulgare* were collected in July 2014 in Chicoutimi, Quebec, QC, Canada. Voucher specimen no. QFA0610840 has been filed at the Louis-Marie herbarium of Laval University, Quebec, QC, Canada. Essential oil was obtained from freshly harvested aerial parts by industrial steam distillation over a 3-h period (Groupe BoréaRessources, Ferland-Boileau, QC, Canada). The essential oil extraction yields depended on the total amount of the raw material. The extracted essential oil was then stored in the dark at a temperature of 4 °C.

### 2.3. GC-MS Analysis

All chromatographic analyses were run on an Agilent 6890N GC (Agilent Technologies, Santa Clara, CA, USA) equipped with a non-polar DB-5 column (Agilent Technologies, Santa Clara, CA, USA) and a polar SolGel-Wax column (30 m × 0.25 mm × 0.25 mm) (Agilent Technologies, Santa Clara, CA, USA) as well as two FID detectors (Agilent Technologies, Santa Clara, CA, USA). The oils were injected in an undiluted (0.1 µL injection volume, split 1:235) and undried state. The temperature program was 40 °C for 2 min, 2 °C·min^−1^ up to 210 °C, and then 210 °C for 13 min. Samples were also injected on an Agilent 7890A GC (Agilent Technologies, Santa Clara, CA, USA) coupled to an Agilent 5975C InertXL EI/CI mass spectrometer (Agilent Technologies, Santa Clara, CA, USA), equipped with a DB-5MS column (Agilent Technologies, Santa Clara, CA, USA) using the same temperature program as above and a split of 1:1000. Compounds were identified from their retention indexes as calculated from even-numbered C8 to C36 alkane standards and/or from MS databases NIST08, HPCH 2205 and custom libraries built from pure compounds (Laboratoire LASEVE, UQAC, QC, Canada). Quantification was derived from the FID detector response on the DB-5 column without any correction factor. All standards were co-injected to validate the identification.

### 2.4. Cell Culture

Human skin fibroblasts WS1 (ATCC CRL-1502), human lung carcinoma A-549 (ATCC CCL-185), human colon adenocarcinoma DLD-1 (ATCC CCL-221), and murine macrophage RAW 264.7 (ATCC TIB-71) were obtained from the American Type Culture Collection (Manassas, VA, USA). The human keratinocytes *HaCaT* cell line was obtained from Dr. Mammoud Rouhabia (Laval University, Québec, QC, Canada). Cells were grown in a humidified atmosphere at 37 °C in 5% CO_2,_ in Dulbecco’s Minimum Essential Medium supplemented with 10% fetal calf serum (Hyclone, Logan, UT, USA), 1 × solution of sodium pyruvate, 1 × vitamins, 1 × non-essential amino acids, 100 IU of penicillin and 100 µg·mL^−1^ streptomycin (Cellgro^®^, Mediatech, Manassas, VA, USA). 

### 2.5. Measurement of Anti-Inflammatory Activity

We evaluated the inhibition NO production by *T. vulgare* essential oil and compounds as described by Legault et al. [23]. Control L-NAME was used as positive control. Murine macrophage RAW 264.7 cells were incubated with essential oil or dissolved compounds in DMSO and then stimulated with 100 ng·mL^−1^ LPS and incubated at 37 °C. After 24 h, we collected the cell-free supernatants and immediately determined the NO concentration using the Griess reaction. We read the absorbance at 540 nm using an automated Varioskan Ascent plate reader (Labsystems, Milford, MA, USA), and quantified the presence of nitrite by comparing with a NaNO_2_ standard curve.

### 2.6. Evaluation of Antioxidant Activity Using Cell-Based Assays

Antioxidant activity was evaluated using the DCFH-DA assay as described by Girard-Lalancette et al. [24]. To assess antioxidant activity, we incubated the human skin fibroblasts WS1 for 1 h with a growing concentration of essential oil or dissolved compounds in DMSO. We then added 100 µL of 200 µM tert-butylhydroperoxide and immediately measured fluorescence as well as again after 90 min later. Measurements were performed on an automated plate reader (Fluoroskan Ascent FL, Labsystems, Milford, MA, USA) using an excitation wavelength of 485 nm and an emission wavelength of 530 nm. Antioxidant activity was expressed as the concentration of extract inhibiting 50% (IC_50_) of DCFH oxidation. 

### 2.7. Evaluation of Antibacterial Activity

We tested the antibacterial activities of *T. vulgare* essential oil and compounds against gram-negative *Escherichia coli* (ATCC bacteria 25922) and gram-positive *Staphylococcus aureus* (ATCC bacteria 25923) using the antibacterial hydrophobic assay as described by Côté et al. [25]. This new method was developed and used in this work as classical methods including disc diffusion and microdilution assays are sometimes inefficient for testing a matrix or compounds of a hydrophobic nature. Briefly, after microorganisms passed 16–18 h at 37 °C in a nutrient broth base (Difco), we transferred 20 µL methanol containing growing concentrations of essential oil and compounds (3.1 to 200 µg·mL^−1^) onto nutrient agar of 96-well plates. We then added bacterial strains having a concentration of 5 × 10^3^ colony forming units (CFU) per mL of nutrient broth. Bacterial suspension without treatment was used as negative control and bacterial suspension plus solvent was tested in parallel to demonstrate the absence of solvent toxicity. The blank consisted of culture medium only and was subtracted from all subsequent measurements of every other well. The 96-well plates were then incubated at 37 °C for 5 h to foster bacterial growth. We then added 100 µL of resazurin sodium salt solution having a concentration of 50 µg·mL^−1^ (Sigma R-2127, St-Louis, MO, USA) to each well. Fluorescence was read on an automated Fluoroskan Ascent FL^TM^ plate reader (Labsystems, Milford, MA, USA) after two hours for *S. aureus* and three hours for *E. coli*. The IC_50_ was determined as the lowest concentration resulting in 50% inhibition of bacterial growth.

### 2.8. Cytotoxic Assays

We plated in 96-well microplates—each containing a 100 µL culture medium—exponentially growing cells of human skin fibroblasts WS1, human *HaCaT* keratinocytes, human lung carcinoma A-549, and human colon adenocarcinoma DLD-1 at a density of 5 × 10^3^ cells per well (Costar, Corning Inc., Lowell, MA, USA). The cells were allowed to adhere for 16 h before treatment. We then treated cells with *T. vulgare* essential oil or dissolved compounds in DMSO. The final concentration of DMSO in the culture medium was 0.5% (*v/v*) to avoid solvent toxicity. After 48 h, we assessed the cytotoxicity using the resazurin reduction test [26]. Fluorescence was measured on an automated Fluoroskan Ascent FL^TM^ plate reader (Labsystems, Milford, MA, USA) using an excitation wavelength of 530 nm and an emission wavelength of 590 nm. Cytotoxicity was expressed as the concentration that inhibited cell growth by 50% (IC_50_). 

## 3. Results and Discussion

The essential oil of *T. vulgare* obtained by steam distillation method was yellow with a distinct odor. The extraction yield obtained after 3 h was 0.23% (*w*/*w*). The refraction index was 1.4797, and the density measured using pycnometer was 0.92396 g·mL^−1^. Table 1 presents the chemical composition of the essential oil as determined by GC-FID analysis. The oil was mainly comprised of oxygenated monoterpenes such as camphor (30.48%), borneol (14.80%), and 1,8-cineole (10.80%). The essential oil also contained camphene (7.29%), bornyl acetate (5.53%), and α-pinene (4.43%). Our results confirm the findings of Collin et al. [17] for *T. vulgare* essential oil from northern Quebec.

We evaluated the biological properties of *T. vulgare* essential oil, including anti-inflammatory, antioxidant, antibiotic, and cytotoxic effects, in vitro using cellular models. Our results for anti-inflammatory activity (Table 2), showed that the *T. vulgare* essential oil inhibited NO production (induced by LPS) with an IC_50_ of 72 µg·mL^−1^. Evaluation of the anti-inflammatory properties of the main compounds present in the oil showed that the most active compound was α-humulene with an IC_50_ of 15 µg·mL^−1^ (Table 2). Fernandes et al. [27] reported that α-humulene reduced rat paw edema that had been induced by carrageenan. It also inhibited COX-2 and iNOS expression. Moreover, the authors concluded that α-humulene might represent an important compound for the management and/or treatment of inflammatory diseases [27]. Other compounds, including α-terpinene, α-pinene, and β-pinene were also effective with an IC_50_ ranging from 30 to 46 µg·mL^−1^ while caryophyllene oxide also inhibited NO production induced by LPS with an IC_50_ of 183 µg·mL^−1^. In a previous study, α-pinene was shown to inhibit NF-κB and JNK activation as well as the expression of iNOS [28]. The anti-inflammatory properties of β-pinene and α-terpinene have also been reported [29,30] as has caryophyllene oxide *in vivo* on mice models [31]. 

The essential oil of *T. vulgare* demonstrated antioxidant activity by significantly inhibiting the oxidation of DCFH induced by tert-butylhydroperoxide with an IC_50_ of 51 µg·mL^−1^ (Table 2). Using in vitro methods including DPPH, ABTS, and FRAP assays, Shaparov et al. [32] also reported a moderate level of antioxidant activity of *T. vulgare* essential oil. Unfortunately, the chemical composition of the oil was not analyzed and the compounds responsible for the activity were not identified. The antioxidant activity of essential oils is often attributed to terpenoids such as carvacrol, thymol, and eugenol [33]. However, our results for *T. vulgare* essential oil showed two compounds, α-pinene and caryophyllene oxide, to be particularly effective having a respective IC_50_ of 3.4 and 6.2 µg·mL^−1^. A moderate level of antioxidant activity for α-pinene, based on a DPPH assay, had been previously reported by Dai et al. [34]. However, to the best of our knowledge, this is the first time the antioxidant activity of caryophyllene oxide has been reported.

Numerous *Tanacetum* species produce essential oils that are known to have antibacterial activity including *T. balsamita*, *T. polycephalum*, *T. parthenium*, *T. longifolium*, and *T. cilicicum* [35,36,37]. Antibacterial activity of *T. vulgare* from Roumania [9] and Slovakia [38] were also reported against gram-positive bacterial strains using the disc diffusion method. Moreover, *T. vulgare* essential oil from Tajikistan was found to be weakly effective against *E. coli* and MRSA [32]. In our study, we used a new antibiotic assay developed to test hydrophobic compounds as described in the Material and Methods section [25]. The results, presented in Table 2, show that *T. vulgare* essential oil was effective against *S. aureus* and *E. coli* with an IC_50_ of 59 µg·mL^−1^ and 241 µg·mL^−1^, respectively. We also tested the antibacterial activity of the main constituents of *T. vulgare* essential oil against *E. coli* and *S. aureus*. Camphor was active against both bacterial strains having an IC_50_ ranging from 22 to 26 µg·mL^−1^. Moreover, caryophyllene oxide and γ-terpinene were also active against *S. aureus* having a respective IC_50_ of 10.4 µg·mL^−1^ and 50 µg·mL^−1^. The antibacterial activity of camphor, caryophyllene oxide, and γ-terpinene has been previously reported in the literature [39,40] although the results are sometimes contradictory. For example, Kotan et al. [41] found camphor to be ineffective as an antibacterial compound. Our use of a new method that is better suited to hydrophobic matrices and compounds [25] may explain the differences in the study outcomes.

We assessed the cytotoxic activity of *T. vulgare* essential oil and its main compounds against human lung carcinoma A-549, colon adenocarcinoma DLD-1, human keratinocytes *HaCaT*, and healthy WS1 cell lines. *T. vulgare* essential oil was slightly cytotoxic against A-549 and WS1 with an IC_50_ higher than 200 µg·mL^−1^ (Table 2). Essential oil was also slightly cytotoxic against human keratinocytes *HaCaT* with an IC_50_ of 199 µg·mL^−1^. Interestingly, DLD-1 (colon adenocarcinoma) was more sensitive to *T. vulgare* essential oil than other cell lines, having an IC_50_ of 105 µg·mL^−1^. To the best of our knowledge, no studies have previously reported the cytotoxicity of *T. vulgare* essential oil against cancer cell lines. The major constituents of camphor, borneol, and 1,8-cineole, were all inactive with an IC_50_ > 200 µg·mL^−1^. However, some minor compounds in the oil, including α-pinene, α-humulene, α-terpinene, β-caryophyllene, β-pinene, γ-terpinene, camphene, caryophyllene oxide, and limonene possessed a moderate cytotoxicity against all cell lines, including the tested cancer cell lines, with an IC_50_ ranging from 28 to 112 µg·mL^−1^. The cytotoxicity of these compounds against various cancer cell lines has also been previously reported [42,43,44,45,46]. 

## 4. Conclusions

Analysis of the chemical composition of *T. vulgare* essential oil from northern Quebec, QC, Canada, demonstrated that it was mainly comprised of oxygenated monoterpene such as camphor, borneol, and 1,8-cineole. The essential oil inhibited NO production (stimulated by LPS and induced by RAW 264.7). The compound α-humulene was, in part, responsible for this anti-inflammatory effect. The antioxidant activity of the essential oil was, in part, due to α-pinene and caryophyllene oxide. Caryophyllene oxide (as well as camphor) was also responsible for antibiotic activity. Finally, *T. vulgare* essential oil inhibited the growth of colorectal cancer cells. In conclusion, *T. vulgare* essential oil possesses interesting biological properties. This study reports, for the first time, the links between the compounds found in *T. vulgare* essential oil and anti-inflammatory, antioxidant, antibacterial, and cytotoxic activities. Additional investigations of *T. vulgare* should be carried out for its potential use for cosmetic or pharmaceutical purposes.

## Figures and Tables

**Table 1:** Chemical composition of *T. vulgare* essential oil from northern Quebec, Canada.

RI DB-5 ^1^	RI S-Wax ^2^	Identified Compounds	Relative CONCENTRATION (%)
903	991	Tricyclene	0.11
911	1010	α-Thujene	0.58
917	1006	α-Pinene	4.43
933	1035	Camphene	7.29
962	1085	Sabinene	2.02
965	1069	β-Pinene	2.51
982	1160	dehydro-1,8-Cineole	0.23
1011	1148	α-Terpinene	0.09
1019	1242	ρ-Cymene	1.20
1023	1172	Limonene	0.16 ^2^
1025	1181	1,8-Cineole	10.80 ^2^
1053	1222	γ-Terpinene	0.29
1061	1443	*cis*-Sabinene hydrate	0.11
1082	1254	Terpinolene	0.09
1096	1405	Filifolone	0.15
1100	1531	Linalol	0.38
1102	1386	α-Thujone	0.08
1109	1405	β-Thujone	3.66
1117	1468	Chrysanthenone	3.76
1132	1614	*trans*-Pinocarveol	0.64
1136	1471	Camphor	30.48
1153	1522	Pinocarvone	0.27
1160	1664	Borneol	14.80
1171	1570	Terpinen-4-ol	0.81
1176	1601	Thuj-3-en-10-al	0.45
1185	1667	α-Terpineol	0.69
1187	1579	Myrtenal	0.11
1190	1752	Myrtenol	0.09
1216	1799	*trans*-Carveol	0.10
1257	1539	*cis*-Chrysanthenyl acetate	0.10
1282	1545	Bornyl acetate	5.53
1352	2113	Eugenol	0.16
1370	1648	Isobornyl propionate	0.75
1391	1880	*cis*-Jasmone	0.08
1411	1556	β-Caryophyllene	0.09
1446	1629	α-Humulene	0.21
1475	1667	Germacrene D	1.13
1482	1674	β-Selinene	0.12
1491	1690	Bicyclogermacrene	0.16
1496	1698	α-Muurolene	˂−Muuro
1505	1701	β-Bisabolene	0.09
1499	1735	Germacrene A	0.09
1503	1721	δ-Amorphene	0.17
1509	1719	γ-Cadinene (1513)	0.15
1519	1742	β-Sesquiphellandrene	0.17
1522	1728	δ-Cadinene	0.63
1536	1865	α-Calacorene	0.19
1573	1922	Caryophyllene oxide	1.13
1593	1976	Ledol	0.65
1597	1970	Rosifoliol	0.07
1622	2015	1-*epi*-Cubenol	0.05
1624	2124	γ-Eudesmol	0.11
1631	2138	τ-Muurolol	0.66
1635	2131	τ-Cadinol	0.19
1641	2171	β-Eudesmol	˂−Eudes
1647	2178	α-Cadinol	0.05
1663	2044	Valeranone	0.07
1713	1880	Pentadecanal	˂entade
Total			99.23

^1^ RI: Retention indices were obtained with a DB-5 column; ^2^ RI: Retention indices were obtained with a SolGel-Wax column.

**Table 2:** Anti-inflammation, antioxidant, antibacterial, and cytotoxic activities of *T. vulgare* essential oil and its main constituents

Compounds	Anti-Inflammation IC_50_ (µg·mL^−1^)	Antioxidant IC_50_ (µg·mL^−1^)	Antibacterial IC_50_ (µg·mL^−1^)		Cytotoxicity IC_50_ (µg·mL^−1^)		
			*E. coli*	*S. aureus*	A549	DLD1	WS1
Essential oil	72 ± 9	51 ± 11	241 ± 13	59 ± 5	232 ± 51	105 ± 18	292 ± 17
1,8-Cineole	>200	>200	>200	>200	>200	>200	>200
α-Humulene	15 ± 2	>200	>200	>200	28 ± 1	43 ± 3	24 ± 3
α-Pinene	30 ± 4	3.4 ± 0.2	nd	nd	35 ± 6	58 ±18	57 ± 4
α-Terpinene	46 ± 4	>200	>200	>200	63 ± 10	73 ± 7	53 ± 12
β-Caryophyllene	>200	>200	>200	>200	55 ± 5	97 ± 6	59 ± 7
β-Pinene	46 ± 9	>200	>200	>200	43 ± 4	49.4 ± 0.3	29 ± 14
γ-Terpinene	>200	>200	>200	50 ± 9	112 ± 15	>200	82 ± 15
p-Cymene	>200	>200	>200	>200	>200	>200	>200
Borneol	>200	>200	>200	>200	>200	>200	>200
Bornyl acetate	>200	>200	>200	>200	>200	>200	>200
Camphene	>200	>200	>200	>200	72 ± 2	75 ± 5	49 ± 1
Camphor	>200	>200	22 ± 1	26 ± 3	>200	>200	>200
Caryophyllene oxide	183 ± 15	6.2 ± 0.5	97 ± 2	10.4 ± 0.9	36 ± 2	43 ± 3	50 ± 4
Limonene	>200	>200	>200	>200	71 ± 6	31 ± 3	53 ± 12
Terpinen-4-ol	>200	>200	>200	>200	>200	>200	>200
Terpinolene	>200	>200	>200	>200	>200	>200	>200

nd: not determined; L-NAME (1 mM), used as an anti-inflammatory positive control, produced an inhibition of NO production of 64.1%; Quercetin, used as an antioxidant positive control, had an IC_50_ of 0.043 ± 0.002 µg·mL^−1^; Gentamycin and chloramphenicol were used as antibiotic positive controls. Gentamycin had an MIC_90_ of 0.9 ± 0.2 µg·mL^−1^ for *E. coli* and 0.0050 ± 0.00005 µg·mL^−1^ for *S. aureus*. Chloramphenicol had an MIC_90_ of 0.9 ± 0.1 µg·mL^−1^ for *E. coli* and 0.73 ± 0.03 µg·mL^−1^ for *S. aureus*; Etoposide, used as a cytotoxic positive control, had an IC_50_ of 2.3 ± 0.2 µM, 2.8 ± 0.4 µM and 19 ± 3 µM for A-549, DLD-1, and WS1, respectively; All the experiments were carried out in triplicate and presented results are representative of at least three different experiments.
